# Influence of Insulin Receptor Single Nucleotide Polymorphisms on Glycaemic Control and Formation of Anti-Insulin Antibodies in Diabetes Mellitus

**DOI:** 10.3390/ijms23126481

**Published:** 2022-06-09

**Authors:** Laura Massarenti, Christina Aniol-Nielsen, Christian Enevold, Henrik Toft-Hansen, Claus Henrik Nielsen

**Affiliations:** 1 Institute for Inflammation Research, Center for Rheumatology and Spine Disease, Copenhagen University Hospital, Rigshospitalet, 2200 Copenhagen, Denmark; laura.massarenti@regionh.dk (L.M.); ciin@novonordisk.com (C.A.-N.); christian.enevold@regionh.dk (C.E.); 2 Clinical Immunogenicity Analysis, Novo Nordisk A/S, 2760 Måløv, Denmark; 3 Immunogenicity Assay Development, Novo Nordisk A/S, 2760 Måløv, Denmark; htoh@novonordisk.com; 4 Section for Oral Biology and Immunopathology, Department of Odontology, Faculty of Health and Medical Sciences, University of Copenhagen, 2200 Copenhagen, Denmark

**Keywords:** diabetes, glycaemic control, anti-insulin antibodies (IAs) single nucleotide polymorphisms (SNPs), insulin receptor

## Abstract

Single nucleotide polymorphisms (SNPs) in insulin and insulin receptor genes may influence the interaction between the two molecules, as may anti-insulin antibodies (IAs), commonly found in patients with type 1 diabetes mellitus (T1D) or type 2 diabetes mellitus (T2D) treated with exogenous insulin. We examined the impact of two SNPs in the human insulin gene (*INS*), rs3842752 and rs689, and two in the insulin receptor gene (*INSR)* rs2245649 and rs2229429, on disease susceptibility, glycaemic control, and IAs formation in 100 T1D patients and 101 T2D patients treated with insulin. 79 individuals without diabetes were typed as healthy controls. The minor alleles of rs3842752 and rs689 in *INS* protected against T1D (OR: 0.50, *p* = 0.01 and OR: 0.44; *p* = 0.002, respectively). The minor alleles of both rs2245649 and rs2229429 in *INSR* were risk factors for poor glycaemic control (HbA1c ≥ 80 mmol/mol) in T1D (OR: 5.35, *p* = 0.009 and OR: 3.10, *p* = 0.01, respectively). Surprisingly, the minor alleles of rs2245649 and rs2229429 in *INSR* associated strongly with the absence of IAs in T1D (OR = 0.28, *p* = 0.008 and OR = 0.30, *p* = 0.002, respectively). In conclusion, the minor alleles of the investigated *INS* SNPs protect against T1D, and the minor alleles of the investigated *INSR* SNPs are associated with poor glycaemic control and the absence of IAs in T1D.

## 1. Introduction

Type 1 diabetes mellitus (T1D) is an autoimmune disease with a prevalence of around 0.2–0.5% in industrialized countries [1]. It is characterized by an autoimmune response against pancreatic islet antigens including insulin, which eventually leads to pancreatic β-cell depletion and, consequently, abolished insulin production resulting in hyperglycaemia [2]. Type 2 diabetes mellitus (T2D) is a metabolic disorder that affects approximately 10% of the world population and is characterized by insulin resistance and progressive pancreatic β-cell dysfunction leading to insulin deficiency [3]. The aetiology of T2D is heterogeneous and comprises a variety of genetic and metabolic factors [4,5]. T1D is treated with exogenous insulin, while insulin is only administered in T2D when non-insulin diabetic drugs are insufficient for the maintenance of euglycaemia. Both T1D and T2D have strong genetic components, including certain *HLA-DRB1* and *HLA-DQB1* alleles, and up to 40 distinct non-HLA susceptibility risk loci [5,6,7,8,9].

The efficacy of exogenous insulin to obtain glycaemic control depends on the amount of endogenous insulin and on the interaction between insulin and its receptor, which may be influenced by polymorphism of both the insulin gene, *INS*, and the insulin receptor (IR) gene, *INSR*, as well as by anti-insulin antibodies (IAs). These are present in approximately 50–80% of patients with diabetes receiving exogenous insulin [10,11] with lower incidence in T2D patients compared to T1D patients [12]. Whether IAs affect the clinical efficacy and safety of therapeutic insulin is controversial (rev. in [13]), but their impact seems limited.

The preproinsulin precursor of insulin is encoded by the *INS* gene, which is located on chromosome 11p15.5 [14]. Several single nucleotide polymorphisms (SNPs) in this gene are associated with T1D [15,16]. These SNPs include the common rs3842752, located in the three prime untraslated region (3’ UTR) of the *INS* mRNA and potentially causing mRNA instability [17], and rs689 located in an intron splice-site [15,18,19,20]. In addition to T1D, rs689 has been shown to associate with T2D [21,22] and the relatively prevalent diabetes subtype, latent autoimmune diabetes of adulthood (LADA) [23,24], which resembles the autoimmune nature of T1D and the metabolic profile of T2D [25,26].

The IR is a key regulator of glucose metabolism found in the membrane of most cells of the human body. Its two isoforms, IR-A and IR-B are results of alternative splicing during transcription of the *INSR* gene located on chromosome 19 [27]. Both isoforms are tetramers composed of two heterodimers linked together by disulphide bonds, and each dimer consists of an extracellular α-subunit with a length of 731 amino acids and a transmembrane β-subunit consisting of 620 amino acids [28]. The α-subunit is responsible for the binding of insulin, while the intracellular domain of the β-subunit mediates signal transduction by virtue of its tyrosine kinase activity [29]. The levels of a soluble form of the insulin receptor (sIR) containing the α subunit and part of the extracellular domain of the β-subunit have been demonstrated to be increased in plasma of patients with T1D or T2D compared to healthy controls and were reported to be associated with hyperglycaemia and hyperinsulinemia [30,31]. Accordingly, injection of purified human α-subunit increases blood glucose levels in mice [32], and transgenic mice secreting soluble α-subunit into the plasma have chronic hyperglycaemia [33], presumably due to sequestering of plasma insulin after binding to the sIR [30].

SNPs in the *INSR* gene have been associated with various phenotypes of insulin resistance [34,35,36,37], including T2D [21,38,39,40]. Reported associations include both intronic and synonymous SNPs and SNPs encoding amino acid changes such as the Val^985^Met, Val^1012^Met, and the Lys^1068^Glu substitutions in the β-subunit [21,38,41]. On the other hand, no SNPs in the gene encoding the IR have yet been associated with T1D, to our knowledge.

We hypothesized that SNPs in *INS* and *INSR* affect not only disease etiology but also glycaemic control, and that changes in the amino acid sequence of insulin or the IR, as a result of SNPs, influence the production of IAs.

To test these hypotheses, we genotyped patients with T1D or T2D, as well as healthy controls, for two common SNPs in *INS*, rs689 and rs3842752, and two common SNPs in the α-subunit encoding portion of *INSR*, rs2245649, and rs2229429, and we examined if these SNPs associate with disease or the circulating levels of glycated hemoglobin (HbA1c), an indicator of glycaemic control, or with sIR levels. Moreover, we analyzed if any of the SNPs associated with the presence of IAs in patients treated with exogenous insulin.

## 2. Results

The study included 100 patients with T1D, 101 patients with T2D, and 79 healthy controls, as shown in Table 1. Patients with T1D were, on average, younger than patients with T2D. The T1D group tended to include more women than the T2D and healthy control groups. Patients with T1D had lower BMI than patients with T2D, while no differences were observed in HbA1c and insulin dosage levels between the two patient groups. Patients with T1D were more frequently IA-positive than patients with T2D. No demographic or clinical data were available for the healthy controls, and sex was inferred through genotyping.

### 2.1. Disease-Associations of Polymorphisms in the Insulin- and the Insulin Receptor Encoding Genes

All study participants were genotyped for rs3842752 and rs689 in *INS*, and r2245649 and rs2229429 in *INSR*. Neither patients with diabetes nor controls deviated significantly from Hardy–Weinberg equilibrium (data not shown).

The minor allele (A) of rs3842752 in *INS* was associated with decreased risk of T1D with an OR of 0.50 (*p* = 0.01) but showed no association with T2D (Table 2). Likewise, the minor allele (A) of rs689 was associated with lowered risk of T1D with an OR of 0.44 (*p* = 0.002), while no association with T2D was observed (Table 2).

The SNPs in *INSR* associated with neither T1D nor T2D (data not shown).

### 2.2. Relationship between SNPs in INSR and Glycaemic Control

We next examined if the selected SNPs associated with glycaemic control, as reflected by visit HbA1c levels. This part of the analysis included 82 patients with T1D and 92 patients with T2D, for whom data on sex, age, BMI, and insulin dosage were all available, so that these factors could be taken into account in multiple logistic regression analyses.

**Figure 1:**
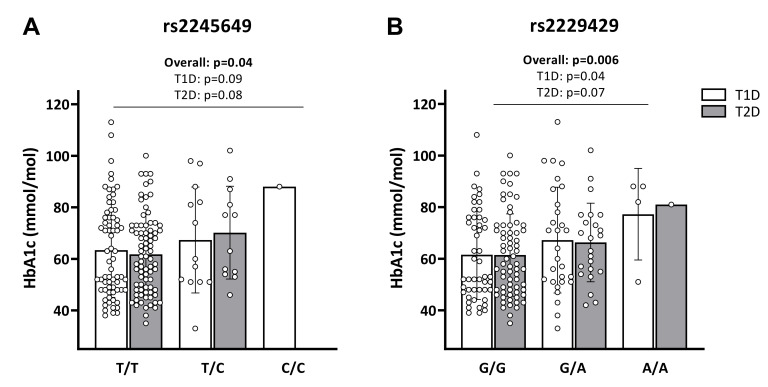
Serum HbA1c levels by *INSR* SNPs genotypes. HbA1c levels (mmol/mol) of patients with T1D (*n* = 82, white bars) and patients with T2D (*n* = 92, grey bars) by genotype distribution of (**A**) *INSR* rs2245649 and (**B**) rs2229429. Trend test was performed with linear regression, adjusted for age, sex, BMI, and insulin dosage.

While we found no association between SNPs in *INS* and glycaemic control (data not shown), we observed an overall allele dosage effect of both rs2245649 (*p* = 0.04) and rs2229429 (*p* = 0.006) in *INSR* on HbA1c levels (Figure 1A,B, respectively), the latter being seemingly driven by T1D for rs2229429 (*p* = 0.04, Figure 1B).

As shown in Table 3, carriage of the minor alleles of rs2245649 in T1D associated significantly with poor glycaemic control, defined as HbA1c ≥ 80 mmol/mol [42,43,44] (OR: 5.35; *p* = 0.009). A similar association was found for rs2229429 (OR: 3.10; *p* = 0.01). In T2D, we observed a similar trend toward an association between carriage of the minor allele (C) of rs2245649 and HbA1c levels ≥ 80 mmol/mol (OR: 3.58; *p* = 0.08), while no association was observed for rs2229429 (Table 3).

When comparing the genotype distribution of rs2245649 and rs2229429 between patients with low (≤53 mmol/mol) and high (≥69 mmol/mol) levels of HbA1c levels at the time of screening, we observed an association between the minor allele of rs2229429 and high HbA1c levels in the T2D group and a trend toward association for rs2245649 as well (Supplementary Table S1). No significant association was found in the T1D group.

### 2.3. Relationship between SNPs in INSR and sIR Levels

We next examined the relationship between circulating levels of sIR and HbA1c levels, on the one hand, and SNPs in *INSR*, on the other hand.

**Figure 2:**
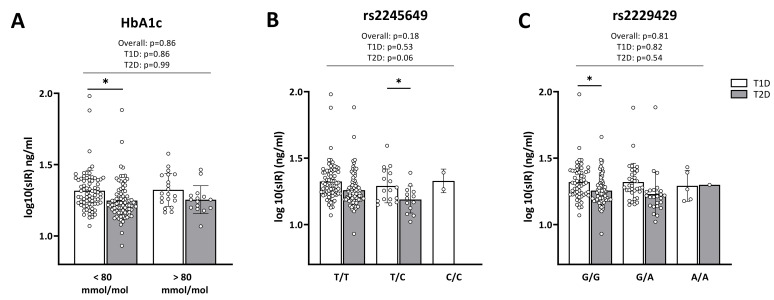
Serum sIR levels by HbA1c levels and *INSR* SNPs genotypes. Shown are serum sIR levels of patients with T1D (*n* = 82, white bars) and patients with T2D (*n* = 94, grey bars) in relation to (**A**) HbA1c levels (mmol/mol), (**B**) *INSR* rs2245649 and (**C**) *INSR* rs2229429. Trend tests and comparisons between T1D and T2D were performed with linear regression, adjusted for age and sex, * *p* ≤ 0.05.

No difference was observed in sIR levels between subjects with HbA1c levels below and above 80 mmol/mol (Figure 2A), nor did sIR levels show significant correlations with *INSR* rs2245649 (Figure 2B) or *INSR* rs2229429 (Figure 2C). However, we did observe a trend towards lower sIR levels in patients with T2D carrying one copy of the minor allele (C) of rs2245649 compared to those heterozygous for the major allele (*p* = 0.06, Figure 2B).

On a separate note, we observed that patients with T2D generally had lower sIR levels than patients with T1D (Figure 2A–C).

### 2.4. Influence of SNPs on Generation of IAs

We have recently analyzed the incidence of IAs in the same cohort of subjects included in this study (Aniol-Nielsen et al., in preparation). We used this information to assess whether the presence of IAs associated with any of the SNPs included in the present study. We observed no such association with the SNPs in *INS* (data not shown).

IAs were significantly less frequent in patients with T1D carrying one or two copies of rs2245649(C) and rs2229429(A) in *INSR* (OR = 0.28, *p* = 0.008 and OR = 0.30, *p* = 0.002, respectively) than in patients homozygous for the major alleles of these SNPs, after correction for age and sex (Table 4). As regards patients with T2D, no association between IA positivity and SNPs in *INS* or *INSR* was observed (data now shown).

**Figure 3:**
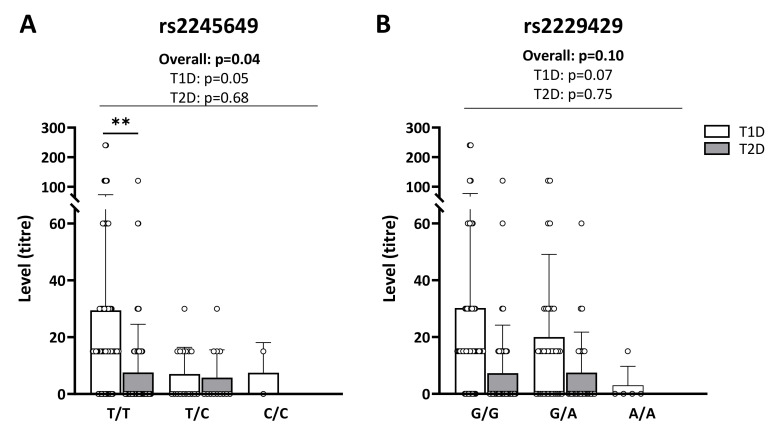
Anti-insulin antibody (IA) titres by *INSR* SNPs genotype. Shown are IA titres for patients with T1D (*n* = 100, white bars) and patients with T2D (*n* = 101, grey bars) in relation to the genotype distributions for (**A**) *INSR* rs2245649 and (**B**) rs2229429. Trend test performed by logistic regression with adjustment for age and sex. Trend tests and comparisons between T1D and T2D by genotype were performed with linear regression, adjusted for age and sex, ** *p* ≤ 0.01.

Accordingly, the patients with T1D showed a borderline-significant inverse correlation between the number of rs2245649 minor alleles (C) and IA titres (Figure 3A), and a similar trend was observed for rs2229429 (T) (Figure 3B). We did not observe any such correlation in patients with T2D.

On a different note, in subjects homozygous for the major allele (T) of rs2245649, we observed higher IAs titres in patients with T1D than in patients with T2D (*p* = 0.01, Figure 3A). Similar trends were observed in subjects carrying one or two copies of the major allele (G) of rs2229429 (*p* = 0.06 and *p* = 0.11 respectively, Figure 3B).

## 3. Discussion

The binding of insulin to the IR plays a key role in the regulation of glucose homeostasis. Here, we studied three factors that may influence this interaction significantly: genetic variation in insulin itself, genetic variation in the α-subunit of the IR, which interacts with insulin, and IAs. We examined if two SNPs in *INS* and two SNPs in *INSR* associated with T1D or T2D, with glycaemic control, and with induction of IAs.

Carriage of the minor alleles (A) of rs3842752 and rs689 in the *INS* gene approximately halved the risk of T1D, confirming previous findings of a role for these SNPs in the pathogenesis of T1D [15,18,19,20]. We found no association between rs3842752 and rs689 in *INS* and T2D, which is in accordance with some previous studies [23,45,46], while other studies have shown an association between rs689 and T2D [21,22], and one study showed associations of both rs689 and rs3842752 with diabetes not classified as either T1D or T2D [47]. Neither of the two SNPs in *INSR* selected for this study associated with T1D nor T2D.

Despite the lack of association of *INSR* SNPs with T1D or T2D, we observed that patients with either diabetes type who were heterozygous for the minor allele (C) in rs2245649 tended to have higher HbA1c levels than patients who were homozygous for the wild type allele. The one T1D patient who was homozygous for the minor allele had poor glycaemic control. Accordingly, in the T1D group the minor allele of the SNP associated with poor glycaemic control defined as HbA1c levels ≥ 80 mmol/mol, which significantly increases the risk of diabetes-related complications, including both micro- and macrovascular manifestations [42,43,44,48]. Increased prevalence of poor glycaemic control was also observed in patients with T1D carrying the minor allele of rs2229429, and increased HbA1c levels were observed in both patients with T1D or T2D carrying the minor allele of this SNP. Furthermore, an analysis performed using 69 mmol/mol as a threshold showed an association between the *INSR* SNPs and HbA1c levels in the patients with T2D, suggesting that the SNPs have an influence on glycaemic control in both groups. SNPs in the portion encoding the α-subunit, like the ones examined here, may reduce the stability and/or affinity of the α-subunit for insulin, causing reduced glucose uptake and, ultimately, increased HbA1c levels. It is also possible that the SNPs increase the ratio between soluble and membrane-bound IR, which may lead to hyperglycaemia because sIR competes with membrane-bound IR for insulin. Supporting this notion, a previous study demonstrated increased sIR levels in the plasma of patients with T1D or T2D compared to healthy controls, and a correlation between sIR and HbA1c levels [30]. However, we observed no correlation between sIR levels and poor glycaemic control or *INSR* SNPs genotype, suggesting that the selected *INSR* SNPs do not influence HbA1c levels solely by regulating the receptor stability but might, instead, affect the transduction of signal to the β subunit. Even though rs2245649 and rs2229429 have been associated with insulin resistance, no studies have previously addressed their functional implications. Although the SNPs selected for this study do not cause amino acid changes, they may, nonetheless lead to the production of less functional proteins by affecting messenger RNA splicing, stability, and structure, as well as protein folding [49,50]. Alternatively, they may reflect the effect of other SNPs located in the *INSR*, and in linkage disequilibrium with those selected in this study.

The role of IAs in diabetes treatment is controversial [12,13,51], but they may inhibit the interaction between insulin and its receptor and thereby cause hyperglycaemia. The ability of IAs to neutralize the effect of insulin likely depends on the affinity between insulin and the IR which, in turn, may be influenced by SNPs in both molecules. We observed that patients with T1D were more likely to produce IAs than those with T2D (71% versus 31%), which likely relates to immune reactivity against endogenous insulin being part of the pathogenesis of T1D [52]. Notably, DQ8, the predominant HLA-type associated with T1D, is capable of binding an amino acid sequence in the β chain of insulin, the InsB_9–23_ peptide [53].

Somewhat surprisingly, we found that the minor alleles of rs2245649 and rs2229429 in the *INSR* were associated with lower frequencies of IA-positive patients with T1D as well as with decreased IA titres, while the frequencies and levels of IAs in patients with T2D were too low to obtain reliable results in this group. A simple explanation for the association in T1D is that IA-producing B cells and the corresponding T-helper cells are, themselves, dependent on insulin-receptor signalling [54,55], and that *INSR* SNPs that lead to a decreased affinity for insulin or decreased functionality of the receptor diminish activation of insulin-reactive B cells and T cells.

Some limitations apply to this study. The relatively low number of participants for a study on genetic polymorphisms only allowed us to study very common SNPs. It would have been of interest to study haplotypes involving SNPs both in the α and β subunits of the IR and to take into account potential interactions between SNPs in *INS* and SNPs in *INSR*. Moreover, info on especially insulin pump dosage was not available for all participants, limiting the analysis of the influence of SNPs on glycaemic control to respectively 82 patients with T1D and 92 patients with T2D. Additionally, a number of factors that impact glycaemic control in diabetes, such as compliance and lifestyle, have not been adjusted for. It should be noted that since the original study objective was to measure IAs in patients with high and low circulating levels of HbA1c, respectively, the patients were selected to have either had HbA1c levels below 53 mmol/mol or above 69 mmol/mol at the time point of screening. Many patients had intermediary levels at the time point of blood sampling, where 23% of the patients with T1D patients and 16% of the patient with T2D patients were poor responders with HbA1c levels ≥ 80 mmol/mol, which is well within the area of 10–30% reported by others [56,57,58]. We find it unlikely that our key findings of an association between SNPs in *INSR* and HbA1c levels and poor glycaemic control, in particular, are affected by the inclusion criteria. Furthermore, the lack of information on age and BMI for the healthy controls did not allow us to adjust for these potential confounders in the analyses of the SNPs’ association with T1D or T2D. In addition, we did not have a measurement of HbA1c levels in the healthy control group. This would have given an indication as to whether our findings also apply to healthy individuals and, consequently, whether analyses of SNPs in *INSR* may be useful in identifying individuals at risk of developing severe diabetes. Further studies addressing this issue are warranted.

In conclusion, our data demonstrate that the two *INSR* SNPs examined are risk factors for poor glycaemic control in T1D and confirm previous findings of an influence of *INS* SNPs on the risk of T1D. Additionally, we show an association between SNPs in the gene encoding the IR and circulating levels of IAs.

## 4. Materials and Methods

### 4.1. Diabetes Mellitus Patients and Healthy Controls

One hundred patients with T1D and 101 patients with T2D were included from the outpatient care clinic at Steno Diabetes Centre Copenhagen. This study is performed with samples from a patient cohort originally collected for a study of IAs (Aniol-Nielsen et al., in preparation) and it includes 40 patients with T1D and 43 patients with T2D with low (≤53 mmol/mol) and 42 patients with T1D and 49 patients with T2D with high HbA1c levels (≥69 mmol/mol) at the time of screening. All data presented in this study were derived from blood collected at the first visit thereafter (except when indicated), thus allowing for continuously distributed HbA1c values. Seventy–nine anonymous blood donors attending the Blood Bank at Copenhagen University Hospital, Rigshospitalet served as healthy controls. The study was approved by the Regional Scientific Ethics Committee, protocol no. H-17016539.

### 4.2. Measurement of Anti–Insulin Antibodies

Serum antibodies against human insulin (Novo Nordisk A/S) were determined in a tiered approach by radioimmunoassay (RIA) as described in [59,60]. The assay sensitivity for IAs at a 5% false-positive level was 4 ng/mL, which complies with regulatory expectations for anti-drug antibody assay sensitivity of 100 ng/mL [59,60]. In the presence of 30 nM human insulin or 30 nM ultra-long–acting analogue, insulin degludec (Tresiba^®^, Novo Nordisk), the assay sensitivity was 6 ng/mL and 50 ng/mL, respectively. Samples were confirmed positive at 37% inhibition of the signal using unlabelled human insulin. Antibody titers were determined as the greatest total dilution, which still produced a signal above the titration cut-off.

### 4.3. Clinical Information

Total insulin dose levels (basal and prandial insulin) and information on insulin regimen were obtained from the patients’ medical records, where applicable (*n* = 174). According to intervals reported in several studies, the threshold for poor glycaemic control was set at HbA1c above or equal to 80 mmol/mol (9.5%), which associates with an increased risk of complications such as retinopathy, nephropathy, and neuropathy as well with the need for a more intensive treatment [42,43,44].

### 4.4. Single Nucleotide Polymorphisms and Genotyping

SNPs in *INS* were selected based on a literature search of previously reported associations with disease susceptibility and included the 3′ UTR variant rs3842752 and the intron splice–site variant rs689 (Table 5). SNPs in *INSR* included the putative intron splice–site variant rs2245649 as well as the exon-synonymous variant rs2229429 (Table 1). All four SNPs were common in populations of European ethnicity with a heterozygote frequency of at least 8%.

DNA was extracted with the Maxwell 16 Blood DNA purification kit on a Maxwell 16 instrument as described by the vendor (Promega, Wisconsin, USA). SNPs were genotyped using an in-house multiplex bead-based assay protocol described in detail in [63]. In brief, allele-specific primers were labelled in an allele-specific primer extension (ASPE) reaction, using SNP–sites amplified by polymerase chain reaction (PCR) as their target sequences. The labelled ASPE–primers were subsequently hybridised to MagPlex-TAG beads (Luminex Corporation, Texas, USA) for detection and counting on the Luminex platform (Luminex Corporation, Texas, USA). For determination of the sex of the healthy controls, and as an additional quality control, we included an in-house test for sex determination based on a six base-pair difference between the X-Y homologous amelogenin genes [64] as part of the multiplex SNP-panel. Eleven control samples with known genotypes, representing the majority of possible genotypes for all included SNPs, were obtained from the Coriell Cell Repository (Camden, New Jersey, USA). These controls, as well as no-template PCR-negative controls, were included in each analyzed plate. To verify the obtained genotypes, approximately 5% of the samples were randomly selected for retyping in a separate analysis. All re-typings were consistent with the first typing.

### 4.5. sIR Measurements

sIR levels were measured in 92 patients with T1D and 94 patients with T2D with the Human Insulin Receptor ELISA kit (BioVendor, Prague, Czech Republic) following manufacturer’s instructions. Serum samples were diluted 5 times and run alongside serial dilutions of a provided standard. A total of 10% of all samples were run twice to confirm measurements obtained in the first runs, and the coefficient of variation was calculated to be 8%.

### 4.6. Statistical Analyses

The ‘hwde’-package in R (R Foundation for Statistical Computing, Vienna, Austria) was used to test for Hardy-Weinberg equilibrium. Associations between SNPs and disease, HbA1c levels ≥ 80 mmol/mol, and IA-positivity were tested using multiple logistic regression with adjustment for age and sex (except the association with presence of disease, only corrected by sex). Correlations between number of carried minor alleles, HbA1c levels, IA titers, and sIR levels were performed with linear regression, corrected by age and sex. Differences in IAs and sIR levels between patients with T1D and patients with T2D were, likewise, evaluated with linear regression with correction for age and sex. Analyses involving HbA1c levels were also corrected by BMI and insulin dosage.

All statistical calculations and analyses were performed in RStudio Version 0.99.902 (RStudio Inc., Boston, MA, USA) using R version 3.3.2 (R Foundation for Statistical Computing, Vienna, Austria), and GraphPad (Prism 8; GraphPad; San Diego, CA) was used for graphical representation of the results. The level of significance was set at *p* < 0.05.

## Figures and Tables

**Table 1:** Demographics and clinical data of the study population.

Variable	Healthy Controls (*n* = 79)	T1D (*n* = 100)	T2D (*n* = 101)	*p*-Value
**Age, years, mean ± SD**	n.a.	48 (15.5)	66.8 (11.6)	<10^−4^
**Female, *n* (%)**	32 (41)	49 (49)	34 (34)	0.07
**BMI, mean ± SD**	n.a.	26.6 ± 4.3	30.8 ± 6.0	<10^−4^
**HbA1c mmol/mol ^§^, mean ± SD**	n.a.	63 ± 18.4	62.5 ± 15.6	0.94
**Insulin dosage IU/kg ^‖^, mean ± SD**	n.a.	0.5 ± 0.3	0.8 ± 1.5	0.10
**IA-positivity, *n* (%)**	n.a.	71 (71)	31 (31)	<10^−4^

T1D: Type 1 diabetes mellitus, T2D: Type 1 diabetes mellitus, BMI: Body mass index, HbA1c: glycated hemoglobin, IAs: anti-insulin antibodies., n.a.: not available. **^§^** Available for 99 patients with T1D. **^‖^** Available for only 82 patients with T1D and 92 patients with T2D. Means between groups were compared with ANOVA, while sex and IA-positivity variables were compared with χ^2^- test.

**Table 2:** Associations between selected polymorphisms in the *INS* gene and T1D (*n* = 100) or T2D (*n* = 101).

	pp	pq	qq	OR	95% CI	*p*-Value
**rs3842752**						
Healthy controls	50	22	7			
T1D	76	23	1	**0.50**	**[0.29;0.87]**	**0.01**
T2D	63	35	2	0.83	[0.50;1.37]	0.46
**rs689**						
Healthy controls	41	31	7			
T1D	73	24	3	**0.44**	**[0.26;0.75]**	**0.002**
T2D	58	33	10	0.90	[0.58;1.41]	0.65

T1D: Type 1 diabetes mellitus, T2D: Type 2 diabetes mellitus, SNP: single nucleotide polymorphism, p: major allele, q: minor allele, OR: Odds Ratio, 95% CI: 95% confidence interval. ^●^ rs3842752 genotype missing for one subject. Values marked in bold indicate 95% CI excluding 1.00, and *p* < 0.05. Logistic regression with adjustment for sex.

**Table 3:** Associations between selected polymorphisms in the *INSR* gene and poor glycaemic control in T1D and T2D.

SNP	Minor Allele	HbA1c < 80 mmol/mol				HbA1c ≥ 80 mmol/mol						
		N	pp	pq	qq	N	pp	pq	qq	OR	95% CI	*p*-value
**T1D**												
rs2245649	C	63	55	8	0	19	13	5	1	**5.35**	**[1.52;18.75]**	**0.009**
rs2229429	A	63	41	21	1	19	8	8	3	**3.10**	**[1.25;7.67]**	**0.01**
**T2D**												
rs2245649	C	77	69	8	0	15	11	4	0	3.58	[0.85;15]	0.08
rs2229429	A	77	57	20	0	15	11	3	1	1.67	[0.50;5.61]	0.41

T1D: Type 1 diabetes mellitus, T2D: Type 2 diabetes mellitus, SNP: single nucleotide polymorphism, HbA1c: glycated hemoglobin, p: major allele, q: minor allele, OR: Odds Ratio, 95% CI: 95% confidence interval. Values marked in bold indicate 95% CI excluding 1.00, and *p* < 0.05. Logistic regression with adjustment for age, sex, BMI, and insulin dosage.

**Table 4:** Associations between selected polymorphisms in the *INSR* gene and the presence of anti-insulin antibodies in T1D.

	Minor Allele	IAs Negative (*n* = 29)			IAs Positive (*n* = 71)					
		pp	pq	qq	pp	pq	qq	OR	95% CI	*p*-value
***INSR***										
rs2245649	C	18	10	1	63	7	1	**0.28**	**[0.11;0.72]**	**0.008**
rs2229429	A	11	14	4	48	22	1	**0.30**	**[0.14;0.65]**	**0.002**

T1D: Type 1 diabetes Mellitus, IAs: anti-insulin antibodies, p: major allele, q: minor allele, OR: Odds Ratio, 95% CI: 95% confidence interval. Values marked in bold indicate 95% CI excluding 1.00, and *p* < 0.05. Logistic regression with adjustment for age and sex.

**Table 5:** Selected SNPs in *INS* and *INSR.*

Ref SNP ID	Gene	Location	Allele Substitution *	MAF	Function	Proposed Clinical Significance
rs3842752	*INS*	chr11:2159843	G > **A**	0.21	3′ UTR variant	T1D [15,18],
rs689	*INS*	chr11:2160994	**A** > T	0.27	Intron splice-site variant	T1D [19,20], T2D [21,22], LADA [23,24]
rs2245649	*INSR*	chr19:7163203	T > **C**	0.08	Putative intron splice-site variant (α-subunit)	Insulin resistance [61]
rs2229429	*INSR*	chr19:7166377	G > **A**	0.19	Exon synonymous	Insulin resistance [61]

MAF: minor allele frequency in Caucasians according to 1000Genomes [62], LADA: Latent Autoimmune Diabetes of Adulthood. * Based on alleles located on the forward strand. Minor alleles are in bold. Genomic locations according to GRCh38.p12 at Genome Browser.

## Data Availability

The datasets generated during and/or analyzed during the current study are available from the corresponding author on reasonable request.

## Supplementary Materials

The following supporting information can be downloaded at: https://www.mdpi.com/article/10.3390/ijms23126481/s1.
