# Supplementary material for: Influence of Insulin Receptor Single Nucleotide Polymorphisms on Glycaemic Control and Formation of Anti-Insulin Antibodies in Diabetes Mellitus
Source: Int J Mol Sci. 2022 Jun 9;23(12):6481. doi: 10.3390/ijms23126481 (PMC9223446; doi:10.3390/ijms23126481)
Supplement: Supplementary file 1 [file ijms-23-06481-s001.zip › ijms-1754446-supplementary.pdf]

Table S1: Associations between selected polymorphisms in the *INSR* gene and poor glycaemic control in T1D and T2D

SNP	Minor allele	HbA1c ≤ 54 mmol/mol				HbA1c ≥ 69 mmol/mol						
		n	pp	pq	qq	n	pp	pq	qq	OR	95% CI	p-value
**T1D**												
rs2245649	C	40	34	6	0	42	34	7	1	1.51	[0.49;4.64]	0.47
rs2229429	A	40	26	13	1	42	23	16	3	1.54	[0.70;3.38]	0.29
**T2D**												
rs2245649	C	43	40	3	0	49	40	9	0	3.17	[0.77;12.94]	0.11
rs2229429	A	43	36	7	0	49	32	16	1	**3.15**	**[1.13;8.79]**	**0.03**

T1D: Type 1 diabetes mellitus, T2D: Type 2 diabetes mellitus, SNP: single nucleotide polymorphism, HbA1c: glycated hemoglobin, p: major allele, q: minor allele, OR: Odds Ratio, 95% CI: 95% confidence interval. Values marked in bold indicate 95% CI excluding 1.00, and p<0.05. Logistic regression with adjustment for age, sex, BMI and insulin dosage.
